# Hypoxia and its therapeutic possibilities in paediatric cancers

**DOI:** 10.1038/s41416-020-01107-w

**Published:** 2020-10-27

**Authors:** Carolina Bernauer, Y. K. Stella Man, Julia C. Chisholm, Elise Y. Lepicard, Simon P. Robinson, Janet M. Shipley

**Affiliations:** 1 Sarcoma Molecular Pathology Team, The Institute of Cancer Research, London, UK; 2 Children and Young People’s Unit, The Royal Marsden NHS Foundation Trust, Surrey, UK; 3 Sarcoma Clinical Trials in Children and Young People Team, The Institute of Cancer Research, London, UK; 4 Division of Radiotherapy and Imaging, The Institute of Cancer Research, London, UK

**Keywords:** Tumour biomarkers, Paediatric cancer, Cancer microenvironment, Cancer therapy

## Abstract

In tumours, hypoxia—a condition in which the demand for oxygen is higher than its availability—is well known to be associated with reduced sensitivity to radiotherapy and chemotherapy, and with immunosuppression. The consequences of hypoxia on tumour biology and patient outcomes have therefore led to the investigation of strategies that can alleviate hypoxia in cancer cells, with the aim of sensitising cells to treatments. An alternative therapeutic approach involves the design of prodrugs that are activated by hypoxic cells. Increasing evidence indicates that hypoxia is not just clinically significant in adult cancers but also in paediatric cancers. We evaluate relevant methods to assess the levels and extent of hypoxia in childhood cancers, including novel imaging strategies such as oxygen-enhanced magnetic resonance imaging (MRI). Preclinical and clinical evidence largely supports the use of hypoxia-targeting drugs in children, and we describe the critical need to identify robust predictive biomarkers for the use of such drugs in future paediatric clinical trials. Ultimately, a more personalised approach to treatment that includes targeting hypoxic tumour cells might improve outcomes in subgroups of paediatric cancer patients.

## Background

Hypoxia, the reduced availability of oxygen compared with its demand in tissues, occurs in multiple solid cancer types. Hypoxic tumours typically exhibit oxygen tensions of <10 mmHg, whereas in normal tissues, the oxygen tension is in the range of 24–66 mmHg.^1^ Tumour hypoxia has been associated with increased tumour aggressiveness, immunosuppression and decreased sensitivity to radiotherapy and chemotherapy.^2^ DNA-damaging free radicals induced by radiation are normally stabilised by oxygen but, in its absence, significantly higher doses of radiation are required to kill cancer cells.^3^ Various mechanisms contribute to the resistance to chemotherapeutic agents in hypoxia,^4^ which include the overexpression of drug efflux proteins^5^ and the induction of autophagy^6^ mediated by the hypoxia-inducible factor (HIF) pathway.

Ultimately, when present, hypoxia leads to poor long-term prognosis in cancer patients,^7^ an observation that provided the rationale for the development of hypoxia-targeted treatment strategies.^8^ The application of such therapies has primarily focused on adult cancers, and any potential benefits have generally been overlooked in paediatric cancers.^9^ Although the prognosis of paediatric cancers has significantly improved over the past 60 years, cancer remains a major cause of death amongst children.^10^ There is therefore an urgent need to improve outcomes for childhood cancer by introducing new treatments that are more targeted and reflect key functional differences between individual patients’ tumours.

In this review, we describe clinically relevant methods to assess tumour hypoxia in children and outline hypoxia-targeted treatment strategies that could be applied in paediatric cancers, such as reducing the extent of hypoxia to restore sensitivity to treatment, designing hypoxia-activated prodrugs (HAPs) to selectively target hypoxic cancer cells and targeting the adaptive mechanisms downstream of hypoxia that enable tumour cells to survive. The need to include biomarker-led patient selection in clinical trials is also highlighted as a key approach.

## Clinical assessment of hypoxia in paediatric cancers

Although numerous hypoxia-targeted treatment strategies have progressed into clinical trials, many of these approaches have not been successfully implemented in the clinic—largely because trial designs have remarkably not included methods to identify and select patients with hypoxic tumours prior to enrolment. Thus, it is necessary to include reliable biomarkers that are predictive of a positive response to hypoxia-targeted treatment strategies in future clinical trials.^11,12^ Table 1 summarises the techniques that are currently clinically available to assess the level of tumour hypoxia, highlighting the advantages and disadvantages of each technique. Although many of the listed advantages apply to both adults and children, the non-invasive nature of some techniques (e.g., hypoxia markers that can be measured in the blood plasma rather than a biopsy) is particularly suitable for children as they may otherwise require anaesthesia and are less tolerant of pain. However, information relating to use of these techniques in children is currently limited. For example, the most direct method to evaluate tumour hypoxia, which involves measuring oxygen partial pressure (pO_2_) with polarographic needles,^13^ has never been performed in children. Moreover, the limited research into hypoxia biomarkers in paediatric cancers has been to primarily determine their prognostic value, rather than understanding their ability to predict the response to standard or hypoxia-targeted treatments. The following section gives a brief overview of the research available on methods to assess hypoxia in children, and discusses the most relevant techniques to implement in the clinic for paediatric patient stratification.

**Table 1:** Currently available methods for assessing the level of hypoxia in tumours of paediatric patients.

O_2_ detection method	Examples	Advantages	Disadvantages	References
Direct O_2_ measurement	Eppendorf pO_2_ electrode	Direct measurement of tissue oxygen level	Invasive long procedure	^13^
			Little-to-no validation in paediatric cancers	
			No longer commercially available	
	Helzel polarographic probe system		Similar disadvantages to Eppendorf precursor, but clinically available	^161^
	Oxford-Optronix OxyLite™ pO_2_ fibre-optic probe	Does not consume oxygen: can measure pO_2_ over long periods of time	Similar disadvantages to Eppendorf and Helzel probes, but clinically available	^162^
		Used in combination with imaging techniques	Long stabilisation time	
Exogenous nitroimidazole hypoxia markers	Pimonidazole hydrochloride, EF5	Detection by histochemical analysis or flow cytometry	Invasive procedure, multiple biopsies preferred	^17,163^
		Microregional (µm scale) distribution of hypoxia	Drug administered in advance	
			Little-to-no validation in paediatric cancers	
Endogenous hypoxia markers	HIF-1, HIF-2, CAIX, GLUT1 and VEGF	Endogenous	Invasive procedure, multiple biopsies preferred	^163^
		Microregional (µm scale) distribution of hypoxia	Expression can vary due to other hypoxia-independent factors (e.g., pH and iron levels)	
		Preclinical/clinical studies in children		
		Cheapest method		
	Circulating osteopontin	Minimally invasive	Surrogate marker for chronic hypoxia without a fully known mechanism	^31^
			Cannot identify tumour hypoxia heterogeneity	
Hypoxia gene signatures	15-gene hypoxia signature, 24-gene hypoxia signature etc.	Only requires one biopsy	Invasive procedure	^33,39,160^
		Accounts for tumour heterogeneity better than a single marker	Difficult to compare different cohorts, even with the same signature	
			Little-to-no validation in paediatric cancers	
Hypoxia imaging techniques	PET/CT (tracer examples: MISO, FMISO, EF5, FAZA, HX4 and 60Cu-ATSM)	Minimally invasive	Little-to-no validation in paediatric cancers	^164^
		Longitudinal evaluation	Requires sedation in young children <5 years	
		Assessment of the entire tumour volume	Expensive	
			Exposure to radiation	
			Limited spatial resolution	
	MRI (examples: DCE-MRI, OE-MRI and BOLD MRI)	Minimally invasive	Little-to-no validation in paediatric cancers	^46^
		Longitudinal evaluation	Requires sedation in young children <5 years	
		Assessment of the entire tumour volume	Expensive	
		No radiation		
		MRI is part of routine assessment of many paediatric tumours		

*BOLD* blood oxygenation-level dependant, *CAIX* carbonic anhydrase IX, *EF5* pentafluorinated derivative of etanidazole, *Cu-ATSM* copper(II)-diacetyl-bis(N(4)-methylthiosemicarbazone), *DCE* dynamic contrast enhanced, *FAZA* ^18^F-fluoroazomycin arabinoside, *FMISO* ^18^F-fluoromisonidazole, *GLUT1* glucose transporter 1, *HIF* hypoxia-inducible factor, *HX4* 3-[^18^F]fluoro-2-(4-((2-nitro-1H-imidazol-1-yl)methyl)-1H-1,2,3-triazol-1-yl)propan-1-ol [^18^F]HX4, *MRI* magnetic resonance imaging, *OE* oxygen enhanced, *O*_*2*_ oxygen, *PET/CT* positron emission tomography/computed tomography, *pO*_*2*_ partial pressure of oxygen, *VEGF* vascular endothelial growth factor.

### Exogenous markers of tumour hypoxia

Some of the most commonly used markers of hypoxia in adult cancers are nitroimidazole compounds. In the absence of oxygen, these bioreductive nitroaromatic compounds undergo reduction to form stable adducts with thiol groups in proteins, peptides and amino acids, which can be detected immunohistochemically.^14^ Examples include pimonidazole hydrochloride and the pentafluorinated derivative of etanidazole, EF5.^15^ Pimonidazole has been used as the standard agent to identify hypoxic areas in tumours in several preclinical studies.^14^ Furthermore, pimonidazole was shown to be a predictive biomarker of the response to hypoxia-targeted carbogen inhalation (a strategy intended to increase tumour oxygenation) in a Phase 3 trial in adult patients with laryngeal cancer.^16^ Although there are no known toxicities that might limit the administration of pimonidazole in children, its potential use as a predictive biomarker of response in children has not been investigated, partly because intravenous administration of these compounds prior to tumour biopsy or surgery would be required. However, an advantage of these exogenous markers is that they are activated in the same conditions as some HAPs, being dependent on both hypoxia and one-electron reductase activity,^17^ which could render them useful as predictive biomarkers of response to HAPs in children.

### Endogenous markers of tumour hypoxia

Tissue hypoxia leads to the activation of HIFs—transcription factors that, in tumours, can mediate the expression of specific genes that orchestrate phenotypic changes, leading to cancer progression and therapy resistance.^18,19^ Differential expression of genes/proteins of the HIF pathway can therefore be used as endogenous markers of hypoxia. The prognostic significance of some of these has been evaluated in paediatric cancers.

In neuroblastoma, the most common extracranial solid tumour in infancy, higher expression of the HIF-1 subunit α (HIF-1α) correlates with low tumour grade and favourable patient prognosis, whilst HIF-2α expression correlates with high tumour stage and unfavourable prognosis.^20^ By contrast, HIF-1α is an unfavourable prognostic factor in bone cancers, including osteosarcomas.^21^ Various paediatric cancers have elevated levels of vascular endothelial growth factor (VEGF), a HIF downstream target, which are associated with worse outcomes.^9^ However, levels of VEGF do not correlate with neuroblastoma stage, or its genotype or phenotype.^20^ Another downstream target of HIF, glucose transporter 1 (GLUT1), is more frequently expressed in neuroblastic tumours (including neuroblastomas) with poor prognosis and unfavourable pathology.^22^ In a separate study, a small cohort of neuroblastoma specimens was evaluated for levels of the HIF downstream targets carbonic anhydrase IX (CAIX) and phosphoglycerate kinase 1 (PGK1); although higher expression of PGK1 has been correlated with strong metastatic behaviour,^23^ this study concluded that CAIX was a stronger negative prognostic factor for survival.^24^ In a small cohort, high levels of CAIX expression in osteosarcoma tumour samples were also shown to be linked with a marginally worse prognosis than low or no CAIX expression, suggesting the potential use of CAIX as a biomarker of high-risk osteosarcoma.^25^ Lastly, lysyl oxidase is another downstream target of HIF that has been shown to be expressed in up to two-thirds of samples from a cohort of patients with retinoblastoma.^26^ Surprisingly, retinoblastoma cell lines were more sensitive to treatment with common chemotherapeutic agents under hypoxic conditions.^26^ This demonstrates the variable significance of these endogenous hypoxia markers in different cancer types, highlighting the importance of investigating them in individual cancers.

Although establishing prognostic significance can support the rationale for targeting hypoxic cells in childhood cancers, it is not enough on its own to ensure that novel agents will be effective in patients. HIF-1α, VEGF, CAIX and GLUT1 were collectively assessed immunohistochemically in tumour tissue microarrays of a small cohort of patients with rhabdomyosarcoma to predict the response to neoadjuvant chemotherapy. The study revealed that the co-expression of three or more of these markers was an independent predictive factor of poor response to chemotherapy.^27^ Further studies must be carried out to understand the potential role of these markers as predictors of response not only to existing treatments, but also to novel hypoxia-targeted treatments.

The endogenous hypoxia marker osteopontin can be detected and quantified in blood samples without the need to carry out a tumour biopsy, enabling the longitudinal assessment of the overall tumour hypoxia burden. Osteopontin is a tumour-associated glycoprotein secreted into the blood plasma that plays a role in several physiological processes, such as tissue remodelling and inflammation;^28^ however, how hypoxia specifically influences the release of osteopontin into the bloodstream remains elusive. Osteopontin levels have been shown to correlate with direct O_2_ measurements, predicting disease progression and response to treatment in some adult cancers.^29,30^ The potential use of osteopontin as a biomarker has been demonstrated in childhood cancers, such as acute lymphoblastic leukaemia (ALL), malignant gliomas^31^ and osteosarcoma,^32^ although further investigation is required for clinical use.

### Hypoxia gene signatures

Several attempts have been made to identify a collection of genes whose expression levels can reflect tumour hypoxia and be used to identify any correlations with disease progression or response to therapy—a hypoxia gene-expression signature. Approximately 30 hypoxia gene signatures have been identified for adult cancers.^33^ A 15-gene signature successfully predicted the clinical outcome and response to hypoxia-targeted therapy in combination with radiotherapy in head and neck squamous cell carcinoma (HNSCC), serving as a prognostic and predictive marker.^34^ This signature was further validated in other cancer types and shown to be the most robust hypoxia gene signature of all those assessed.^35^ Another hypoxia-related 28-gene signature was validated in prostate cancer as a prognostic and predictive biomarker for assessing the benefit of hypoxia-modifying therapy.^36^ A 24-gene hypoxia signature has been defined in bladder cancer patients^37^ and another in soft-tissue sarcoma patients.^38^

The reliability of a single biopsy sample to reflect the hypoxic status of a tumour was compared with multiple biopsy samples using gene signatures, including three of the signatures mentioned above, or single gene markers of hypoxia. The study demonstrated the ability of a gene signature from a single biopsy sample to correctly classify a tumour as more or less hypoxic, although small variability between biopsy samples remains unavoidable.^39^ Currently, hypoxia gene signatures are derived from single biopsy samples, and for this method to be clinically relevant in children, it is essential that a single biopsy sample is representative of the entire tumour.

The outlook for identifying and using hypoxia gene signatures in paediatric cancers is encouraging, although data are currently limited. In 2016, a set of nine genes identified in neuroblastoma tumour samples and hypoxic neuroblastoma cell lines was shown to be associated with aggressive neuroblastomas,^40^ thus representing a potential novel hypoxia gene signature for a paediatric cancer.

### Hypoxia imaging

Non-invasive imaging methods to repeatedly and rapidly quantify the extent and heterogeneity of hypoxia within an individual tumour in vivo would offer a clinical benefit in personalised treatment planning.^41^ There is a pressing need for imaging biomarkers not only to identify patients with hypoxic tumours, but also to map the degree and distribution of hypoxia to optimise radiotherapy planning and to assess the effects of hypoxia-modifying approaches for therapeutic gain.^42,43^

Magnetic resonance imaging (MRI) provides a non-invasive approach for monitoring cancer. MRI exploits the magnetic moment of protons in water within the tissue to create high-resolution anatomical images with exquisite soft-tissue contrast. As non-ionising radiation is used, MRI lends itself well to repeat investigations. Image acquisition can be sensitised to numerous independent contrast mechanisms, from which parametric maps can be calculated and used to provide additional information on tumour structure and function. Such multiparametric MRI strategies can thus be used to evaluate intratumoural heterogeneity and its relationship to tumour progression and treatment response.^44^

Several MRI techniques are being actively investigated for delivering translational imaging biomarkers of hypoxia.^45,46^ One approach, intrinsic susceptibility MRI (IS-MRI), exploits the paramagnetic properties (the ability to induce internal magnetic fields due to unpaired electrons) of deoxyhaemoglobin in erythrocytes to create image contrast. Deoxyhaemoglobin therefore acts as an intrinsic, blood oxygenation-level-dependent contrast agent (thus, this approach is also known as BOLD MRI). IS-MRI investigations quantify native tumour R_2_* and/or the change in R_2_* (ΔR_2_*) induced by respiratory challenge with a high-oxygen content gas. Hyperoxia-induced decreases in R_2_*, which reflect changes in blood oxygenation due to a change in haemoglobin saturation, have been shown to be associated with improved tumour oxygenation.^47,48^ A series of preclinical IS-MRI studies performed in two separate laboratories using syngeneic rat rhabdomyosarcoma models have shown that this tumour type exhibits a relatively high native R_2_* and a marked hyperoxia-induced reduction in R_2_*, consistent with a hypoxic phenotype.^49,50^ These studies also highlight the potential of IS-MRI to provide predictive imaging biomarkers of rhabdomyosarcoma response to radiotherapy. Investigation of tumours arising in transgenic mouse models of neuroblastoma revealed extremely high native R_2_* values and marked hyperoxia-induced ΔR_2_*, but with negligible evidence of hypoxia assessed histologically.^51^ The IS-MRI data are consistent with aberrant haemodynamic vasculature typically ascribed to this paediatric cancer. Collectively these preclinical studies highlight that the relationship of native R_2_* to tissue hypoxia can vary according to the underlying histology.^46^ This is important because the underlying histology influences the level of hypoxia in a tumour.

**Fig. 1:**
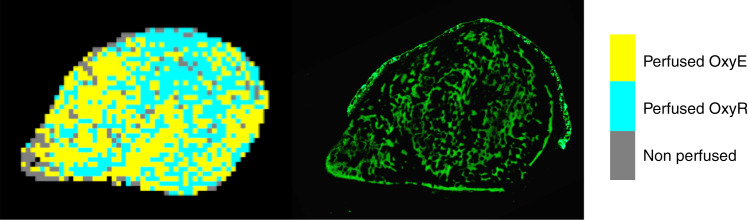
OE-MRI mapping of hypoxia in a rhabdomyosarcoma cell line model. The image shows a RH41 rhabdomyosarcoma cell line xenograft, in which perfused voxels that are refractory to hyperoxia-induced changes in R_1_ (perfused OxyR), are coloured blue, perfused and oxygenated voxels (perfused OxyE) are coloured yellow and necrotic voxels (non-perfused) are coloured grey (image on the left). An aligned section stained for pimonidazole adduct formation in green was used as an established marker to map tumour hypoxia (image on the right). Significant areas of hypoxia are mapped.

An alternative emerging approach, oxygen-enhanced MRI (OE-MRI), relies on quantifying hyperoxia-induced signal changes—the longitudinal MRI relaxation rate R_1_ (s^−1^)—caused by excess oxygen molecules dissolved in blood plasma and interstitial fluid.^52^ In oxygenated tissues, hyperoxia induces a change in the signal intensity,^53^ whereas in hypoxic tissue, oxygen molecules that have been inhaled preferentially bind to deoxygenated haemoglobin, rather than dissolving in blood plasma and interstitial fluid, resulting in unchanged signal intensities. Comparison with image-aligned tissue sections stained for pimonidazole showed that the perfused tumour areas refractory to hyperoxia-induced changes were hypoxic.^53^ Preclinical and clinical studies have demonstrated that the volume of oxygen-refractory voxels can be used to map and quantify hypoxic fractions across a range of tumour types.^54–57^ OE-MRI revealed extensive tumour subregions that were refractory to oxygen-induced signal intensity changes in a syngeneic rat rhabdomyosarcoma model, again consistent with the presence of hypoxia.^49,50^ Preliminary OE-MRI investigations in RH41 rhabdomyosarcoma xenografts also suggest a strong hypoxia signature in this tumour type (Fig. 1).

MRI is used routinely in healthcare, and both IS-MRI and OE-MRI have been implemented on standard clinical scanners and used to assess tumour hypoxia in adult patients.^55,57,58^ Whilst the application of MRI protocols in children can be challenging due to the logistical challenges involved, such as having to sedate/anaesthetise younger patients to undergo the exam, rapid mapping of tumour hypoxia with IS-MRI and OE-MRI, which both rely on endogenous contrast mechanisms, is totally non-invasive and relatively straightforward to incorporate into existing clinical imaging protocols. We predict that within the next 5–10 years, this is likely to enhance imaging-embedded investigations associated with clinical interventions that may ultimately improve the outcome for paediatric patients.

## Targeting hypoxia in paediatric cancers

Hypoxia is a potentially targetable characteristic of most locally advanced solid tumours.^59^ Many early-phase clinical trials in children rely on successful adult Phase 3 trials; however, it is important to consider—from a therapeutic perspective—that differences exist in the aetiology between adult and paediatric cancers.^60^ Nevertheless, hypoxia has been shown to be important in several paediatric cancers. As outlined above, the presence of endogenous hypoxia markers has a negative impact upon paediatric cancer prognosis. Hypoxia is also likely to be a treatment-limiting factor in the most prevalent paediatric cancers, such as leukaemia, brain cancer and sarcomas: for instance, chemoresistance was demonstrated to be promoted by hypoxic conditions in ALL cell lines via the modulation of cell death signalling pathways.^61^ Medulloblastoma cell lines under hypoxic conditions have a significant reduction in therapy-related DNA damage after treatment with etoposide and radiotherapy.^62^ Similarly, rhabdomyosarcoma and Ewing’s sarcoma cell lines develop resistance to treatment-induced apoptosis in hypoxic conditions.^63^ Therefore, the inclusion of hypoxia-targeted approaches in treatment regimens has the potential to benefit paediatric cancer cases.

**Fig. 2:**
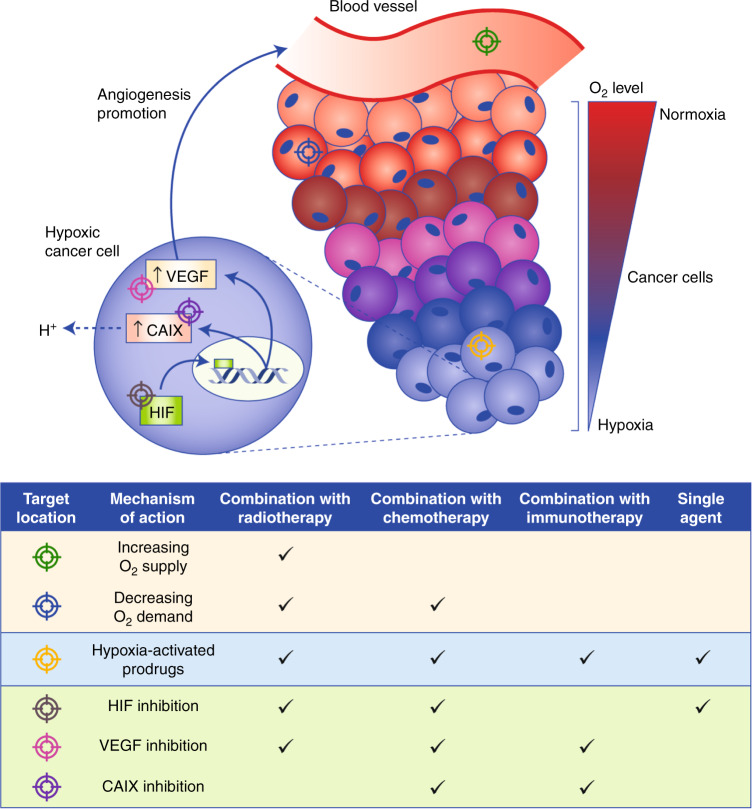
Hypoxia-targeted treatment strategies. Key interactions between tumour hypoxia and the tumour vasculature are depicted by the blood vessels and cancer cells of a solid tumour. The levels of oxygen (O_2_) in cancer cells decrease with increasing distance from the blood vessel. In hypoxic cancer cells, hypoxia-inducible factor (HIF), a transcription factor, enters the nucleus and upregulates the expression of target genes, such as vascular endothelial growth factor (VEGF), which promotes angiogenesis, thus improving the blood supply to promote tumour progression, and carbonic anhydrase IX (CAIX), which regulates the acidification of the extracellular tumour microenvironment (TME). Counteracting the effects of hypoxia in a solid tumour for therapeutic benefit can be achieved by targeting distinctive areas of the tumour with therapies that have different mechanisms of action, as depicted: (i) increasing the supply of O_2_ to cancer cells through surrounding blood vessels, (ii) decreasing the O_2_ demand in well-oxygenated perihypoxic cells, thereby increasing O_2_ availability for hypoxic cells, (iii) using inactive prodrugs that are activated by enzymes present specifically in hypoxic cells, (iv) directly inhibiting HIF and its downstream effects that enable hypoxic cells to adapt to hypoxia and inhibiting HIF downstream targets, such as (v) VEGF or (vi) CAIX. These treatment strategies can be used in combination with other therapies, such as radiotherapy and/or chemotherapy, or immunotherapy, or as single agents, for optimal therapeutic benefit.

To date, three main hypoxia-targeted strategies have been developed. The first involves increasing the level of oxygen in the tumour microenvironment (TME). Alternatively, hypoxia can be exploited so that hypoxic cancer cells are selectively targeted. Finally, the adaptive mechanisms that enable tumour cells to survive in hypoxia can be targeted.^8^ These strategies are summarised in Fig. 2. The average level of oxygenation in peripheral tissues is 6.1%,^64^ whereas in solid tumours, median oxygen levels can vary between 0.3^65,66^ and 4.2%.^67^ As areas of pathological hypoxia are heterogeneously distributed throughout tumours^13^ between areas of physiological oxygenation, it might be necessary to combine hypoxia-targeting drugs with existing therapies—chemotherapy, radiotherapy and immunotherapy—to effectively treat all regions.^18^

## Hypoxia-targeted treatment strategy 1: increasing oxygen availability in the hypoxic tumour microenvironment

Tumour cells evolve in parallel with various components of the TME, including the extracellular matrix and cells of the vascular, stroma and immune systems. The influence of hypoxia on the TME of paediatric cancers is documented in multiple studies.^68–71^ Hypoxia features in the microenvironment of bone marrow, and increased HIF-1α expression and the acquisition of a glycolytic phenotype (through AKT/mammalian target of rapamycin [mTOR] signalling activation) was demonstrated in co-cultures of leukaemic cells with bone marrow-derived mesenchymal stem cells under hypoxic conditions.^68^ Medulloblastoma cell lines upregulate HIFs under hypoxic conditions, and a decrease in cellular proliferation was observed in response to knockdown of the *HIF1A* gene.^69^ In studies of cells derived from primary Ewing’s sarcoma family tumours, the hypoxic microenvironment was shown to upregulate expression of the EWS–FLI1 fusion protein^70^ and to increase cellular invasion in vitro.^71^ Thus, the interactions between the various components of a tumour influence the tumour’s ability to proliferate and metastasise, and its response to therapy.^59^ Treatments that improve oxygen availability to hypoxic cancer cells in the TME aim to sensitise tumours to existing therapies.

### Increasing oxygen delivery in combination with radiotherapy

The first correlation between the availability of oxygen and radiosensitivity was discovered in the early twentieth century whereby greater inhibition of root growth during germination was observed with aerobic seeds compared with anaerobic seeds after irradiation.^72^ Later studies conducted in irradiated, tumour-bearing mice, demonstrated greater tumour regression in mice inhaling 100% oxygen rather than ambient air.^73^ Thomlinson and Gray were the first to infer the presence of hypoxic cells in human lung cancer sections and comment on their implications for radiotherapy.^74^

The earliest attempts to increase tumour oxygenation during radiotherapy included the use of hyperbaric oxygen, blood transfusions and administration of erythropoietin (to stimulate the generation of red blood cells from stem cells). These therapies never became standard-of-care treatment, however, due to conflicting efficacies in clinical trials.^75^

A further method to increase tumour oxygenation is the inhalation of carbogen—usually a mix of 5% carbon dioxide and 95% oxygen gas—during radiation treatment. Carbogen inhalation proved its efficacy in Phase 3 trials, significantly improving 5-year regional control (93% compared with 86% for radiotherapy alone) in adult patients with HNSCC.^16^ Improved 5-year overall survival (OS) rates (50% compared with 39%) were also reported in adult patients with bladder cancer.^76^ In both cases, carbogen was co-administered with nicotinamide, which reduces fluctuations of blood flow.^75^ Retrospective studies using hypoxia gene signatures for both trials confirmed that hypoxic tumours responded more optimally than normoxic tumours to hypoxia-modifying treatment.^37,77^ Despite some reports of breathing discomfort alongside carbogen inhalation, compliance in adults is high;^78^ in children, carbogen inhalation per se is reported to be safe and feasible without any significant compliance issues.^79–81^ It is, however, foreseeable that this therapy would be more suitable for older children, or toddlers and babies that have already been sedated for radiotherapy. In a clinical trial that combined carbogen inhalation with radiotherapy for the treatment of high-grade paediatric gliomas, no increase in survival over radiotherapy alone was noted.^82^ It is important to note, however, that tumour hypoxia was not evaluated in this small trial, and so the need to select for compliant patients with hypoxic tumours for paediatric cancer clinical trials remains untested.

### Decreasing oxygen consumption in combination with radiotherapy and/or chemotherapy

Decreasing the oxygen consumption of perihypoxic cells in tumours is another approach to modify the level of oxygen availability.^83^ Mathematical modelling has shown that decreasing oxygen consumption in well-perfused areas is more effective at increasing oxygen availability in hypoxic areas than attempts to increase oxygen supply in these areas.^84^ The antidiabetic drug metformin alleviates hypoxia by inhibiting oxidative phosphorylation through inhibition of mitochondrial complex I, thereby reducing the oxygen consumption rate (OCR) in cancer cells.^85^ A 2018 Phase 1 trial of advanced HNSCC evaluating the addition of metformin to standard cisplatin and radiotherapy showed promising results (relative to historical control rates^20^) with 2-year OS rates and progression-free survival (PFS) rates of 90 and 84%, respectively. A Phase 2 trial for this combination in adult cervical cancer (NCT02394652) is currently at the recruitment stage.

Preclinical evidence supporting the use of metformin in childhood cancers is variable. Metformin successfully restored chemosensitivity to cisplatin in hepatoblastoma cells under hypoxia.^86^ Conversely, the inhibitory effects of metformin on tumour growth were reduced by hypoxia in childhood sarcoma xenografts.^87^ The disparity in results may be due to the inherent differences between hepatomas and sarcomas and/or the variabilities in experimental details, including drug concentrations and methods used to induce hypoxia. The pleiotropic effects of metformin on cancer cells are also apparent in adults, with inconsistent outcomes in adult clinical trials, too.^88^ Nonetheless, metformin is currently being tested in a Phase 1 clinical trial that aims to evaluate escalating doses of the agent in combination with a backbone therapy of vincristine, irinotecan and temozolomide in children with recurrent and refractory solid and brain tumours (NCT01528046).^89,90^

Atovaquone is an antimalarial drug found to reduce OCR—to an even greater extent than metformin—through the inhibition of mitochondrial complex III. Clinically achievable doses of atovaquone are capable of reducing OCR in vitro and alleviating tumour hypoxia in vivo, and consequently increase the radiosensitivity of various cancer types.^91^ Metformin was also tested; however, the dose required to achieve a similar reduction in OCR is not achievable in patients. Atovaquone can therefore be added to standard-of-care treatment without additional severe toxicities in patients.^91^ These results have prompted a clinical trial (NCT02628080) for non-small- cell lung cancer in which immunohistochemical, serological and imaging of hypoxia biomarkers are evaluated in addition to clinical outcomes.^83,91^

Atovaquone is successfully used in children with leukaemia as a prophylactic approach against *Pneumocystis carinii*.^92^ Repurposing safe, commonly prescribed drugs is a superficially favourable approach, as the limitations of testing new agents in children exceed those for adults; known safety and toxicity profiles shorten the timeline and reduce the number of patients required—already scarce to start with—to obtain approval for a novel indication in children.^93,94^ Ethically, these patients must receive the highest standard of care and some patients/parents are not willing to accept the safety risks involved with Phase 1 testing.^93^ Together with the lack of commercial interest to develop new drugs with the sole indication of treating rare paediatric cancers,^93,94^ these factors support repurposing atovaquone in children, although preclinical evidence must be generated to support this combination before clinical trials commence.

## Hypoxia-targeted treatment strategy 2: targeting hypoxic tumour cells directly

Oxygen concentrations are frequently markedly lower in malignant tissues compared with their non-malignant surroundings, rendering hypoxia a targetable feature in tumours. Bioreductive prodrugs have been developed to exploit the endogenous expression of human cellular oxidoreductases in hypoxic tumour cells, such that only in the absence of oxygen, can oxidoreductases reduce the prodrug and convert it into an unstable prodrug radical. This in turn can then be further reduced or fragmented into a toxic metabolite. Hence, the conversion of the prodrug into its toxic metabolite is activated under conditions of hypoxia, but inactivated in the presence of oxygen:^15^ hence the name hypoxia-activated prodrugs (HAPs). HAPs can be divided into five main subgroups: nitroaromatic compounds (e.g., nimorazole, PR-104 and evofosfamide), aromatic N-oxides (e.g., tirapazamine), aliphatic N-oxides, quinones and metal complexes. Of these subgroups, nitroaromatic compounds and aromatic N-oxides have progressed the furthest in clinical development and have been preclinically tested.

### HAPs as single-agent treatments

As a single-agent therapy, HAPs must target both the hypoxic and normoxic components of tumours. This can be achieved through the active bystander effect, whereby the active metabolite of the HAP is not only generated in hypoxic areas, but also diffuses into neighbouring normoxic cells.^15^ For example, an active bystander effect was observed using PR-104 in a three-dimensional spheroid model combined with mathematical modelling.^95^ Single-agent activity of PR-104 in mouse xenograft models^96^ and activation in oxic cancer cells by human aldo–keto reductase 1C3^97^ have both been demonstrated. Single-agent efficacy of evofosfamide is due to very high plasma concentrations of the drug, achievable in patients. This provides significant bioreductive activation despite partial inhibition by oxygen.^98^ Evofosfamide has single-agent efficacy in vitro, and in mouse xenograft tumour models of adult nasopharyngeal carcinomas,^99^ as well as in patient-derived xenograft models of HNSCC.^100^

Although single-agent activity might be desirable, the activation of prodrugs by intermediate levels of oxygen could limit their clinical use due to the potential risk of toxicities in healthy tissues undergoing physiological hypoxia, such as in the liver and brain.^64^ Predicting off-target toxicity will therefore be fundamental for the implementation of these agents. The spatially resolved pharmacokinetic/pharmacodynamic (SR-PK/PD) model is potentially a valuable tool for predicting off-target toxicity.^101^

### HAPs in combination with radiotherapy

Early members of the class of nitroaromatic compounds were shown to mimic the radiosensitisation caused by oxygen in non-hypoxic tissues, and were therefore originally developed to be radiosensitisers, so-called oxygen mimetics.^102^ The first available nitro-based HAPs, such as misonidazole, proved to be clinically disappointing, however, as undesirable side effects limited the dose.^103^ Nimorazole was soon demonstrated to be less toxic and to have similar radiosensitising properties at clinically achievable doses.^104^ Nimorazole significantly improved the efficacy of radiotherapy in adult HNSCC without major side effects, leading to its routine clinical use in Denmark.^105^ Consequently, a UK-based Phase 3 clinical trial was set up to assess the benefit of using nimorazole during radiation treatment of patients with HNSCC, the results of which are yet to be published.^106^ These studies suggest that nimorazole could be a potential therapy for paediatric cancers, although this notion remains untested to date. Although tolerance in adults due to nausea and vomiting is an issue, there are no obvious concerns for the use of nimorazole in children and it is less toxic than similar compounds, such as misonidazole and etanidazole.

**Fig. 3:**
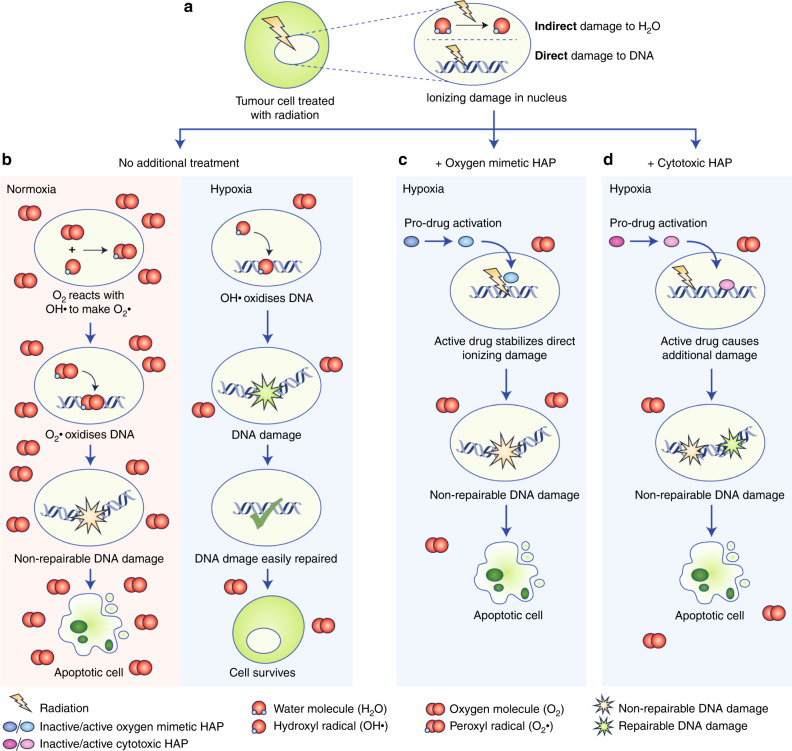
Schema: modes of action for hypoxia-activated prodrugs (HAPs). A schematic diagram that compares the differing stages of events within the tumour cell nucleus following treatment with radiation in the presence and absence of an oxygen mimetic HAP or a cytotoxic HAP in hypoxia. **a** During radiation, a high-energy electron indirectly causes DNA damage by impinging on a water molecule (H_2_O) to form a reactive hydroxyl radical (OH·). Radiation also interacts directly with DNA, producing radiation-induced DNA damage. **b** In normoxia, free oxygen molecules (O_2_) interact with OH·, promoting the formation of a peroxyl radical (O_2_·), that interacts with DNA, causing non-repairable damage resulting in cellular apoptosis. In hypoxia, the OH· radiation-induced DNA damage is repaired to its original state preventing cell death. **c** In hypoxia, an oxygen mimetic HAP (e.g., nimorazole) converts into its activated form, stabilising the radiation-induced DNA damage to promote apoptosis. **d** A cytotoxic HAP (e.g., evofosfamide) prodrug is also converted into its active form in hypoxia, consequently inducing DNA damage, independently of the radiation-induced DNA damage, leading to apoptosis.

The development of cytotoxic DNA-targeting bioreductive agents (e.g., PR-104, evofosfamide and tirapazamine) soon followed the development of oxygen mimetics.^102^ Both drug classes constitute HAPs as they depend on the absence of oxygen for activation by endogenously expressed oxidoreductases. However, cytotoxic DNA-targeting HAPs cause DNA damage, whereas oxygen mimetics merely stabilise existing radiotherapy-induced DNA damage, as oxygen does.^107^ The different modes of action of HAPs are illustrated in Fig. 3. A Phase 3 clinical trial in patients with cervical carcinoma did not show any added benefit on OS or PFS upon the addition of tirapazamine to cisplatin and radiotherapy.^108^ A Phase 3 trial testing this same combination in HNSCC patients also failed to show any improvement in OS.^109^ However, neither of these trials selected patients with hypoxic tumours for treatment with tirapazamine, and it was suggested in both cases that biomarker-led patient selection would improve the results. Evofosfamide was shown in 2019 to sensitise oesophageal cancers to radiotherapy without increased levels of toxicity in vivo.^110^ Similar results were obtained when this combination was tested in a syngeneic rat rhabdomyosarcoma model. The authors noted that the efficacy of treatment was dependent on the tumour oxygenation status, which was evaluated using [^18^F]HX4 hypoxia PET imaging.^111^ Again, including hypoxia biomarkers in clinical trials could enhance the efficacy of these treatments.

### HAPs in combination with chemotherapy

The majority of clinical trials of cytotoxic HAPs have involved combination with chemotherapeutic agents. In this approach, non-hypoxic cells within a tumour are expected to be primarily targeted by the cytotoxic activity of chemotherapeutic drugs, whilst hypoxic cells are targeted by HAPs, thereby treating both cellular populations of the tumour.^112^ The combination of evofosfamide with topotecan, a topoisomerase inhibitor, was investigated in cell line models of neuroblastoma and rhabdomyosarcoma. In contrast to single agents, the combination treatment led to complete tumour regression and improved response in the neuroblastoma and rhabdomyosarcoma models, respectively. Moreover, the combined treatment was shown to induce tumour cell apoptosis in both hypoxic and non-hypoxic regions.^113^

Tirapazamine is clinically safe in children,^114^ and has been investigated as a promising drug candidate for paediatric cancers. Unfortunately, however, the effectiveness of tirapazamine in treating paediatric rhabdomyosarcoma could not be demonstrated in the absence of predictive patient stratification.^115^ Similarly, in adults, large, randomised Phase 3 trials failed to achieve positive results, despite promising Phase 1 and 2 trials for tirapazamine and evofosfamide.^11,12^ For example, the TH CR-406/SARC021 trial in patients with locally advanced, unresectable or metastatic soft-tissue sarcoma, based on promising Phase 2 trial results,^116^ failed to show any benefit from the addition of evofosfamide to doxorubicin.^117^ The level of tumour hypoxia prior to the administration of hypoxia-targeted therapies was not evaluated in any of these trials. Although the combination of HAPs with chemotherapy remains a promising therapy for paediatric cancers, again, reliable biomarkers must be identified to effectively predict the response to HAPs and to identify which patients are most likely to benefit from these therapies.

## Hypoxia-targeted treatment strategy 3: targeting the downstream effects of tumour hypoxia

The activation of HIFs in hypoxic tissues promotes angiogenesis, metabolic reprogramming and a stem-cell-like phenotype, which collectively increases tumour invasion and metastasis.^118^ HIF-mediated resistance to chemotherapy is linked to the induction of autophagy, defective apoptosis, reduction of DNA damage and overexpression of drug efflux proteins.^76^ In hypoxic conditions, resistance to radiotherapy is primarily due to the lack of oxygen to stabilise radiation-induced free radicals.^119^ Additionally, increased HIF activity has a protective effect on the tumour vasculature.^120^ Higher HIF-1 expression levels after radiation lead to the secretion of VEGF, which inhibits apoptosis in endothelial cells, thereby reducing the vascular damage normally caused by radiation.^121^ The discovery of this key regulatory pathway has led to several attempts to inhibit HIF and its downstream targets.^122^

### HIF inhibition

Geldanamycin, a small molecule that indirectly inhibits HIF activity, showed promising results in paediatric Phase 1 trials. However, dose-limiting toxicities were observed in one of the two trials.^123,124^ HIF-1 inhibitors such as PX-478 and NSC-134754 were also included in adult Phase 1 clinical trials,^9^ but as yet, have not been introduced in the paediatric cancer setting. Selectively inhibiting HIF has proved to be a challenge, and no small-molecule HIF-1 inhibitors have so far been clinically approved, most likely because they lack the desired specificity.^125^

HIF-2α has been described to have an important role in tumour progression,^126^ and is now emerging as a potential target. A Phase 3 trial for adult clear-cell renal cell carcinoma is planned with the second-generation HIF-2α inhibitor, PT2977.^127^ A first-generation HIF-2α inhibitor, PT2385, was unable to inhibit downstream signalling and had no effect on the response to chemotherapy when tested in paediatric neuroblastoma patient-derived xenografts, implying that this may not be an appropriate target in neuroblastoma.^128^ Furthermore, another study found that HIF-2ɑ demonstrates tumour-suppressive properties in neuroblastoma tumours treated with the DNA-demethylating drug 5-Aza-deoxycytidine (AZA) and retinoic acid.^129^

Nonetheless, interest in HIF inhibition as a therapeutic strategy for paediatric cancer continues, particularly for paediatric brain tumours. Blocking HIF-1α transcriptional activity with 2-methoxyestradiol increased the cytotoxic activity of cyclophosphamide and ifosfamide in medulloblastoma cells.^130^ Combining the inhibition of histone methyltransferase enhancer of zeste homologue 2 (EZH2) with HIF inhibition has also been proposed as a potential new treatment strategy for paediatric high-grade gliomas.^131^ Acriflavine, a safe, small molecule with HIF-inhibiting properties, was shown to downregulate HIF-mediated pathways and induce cell death in both in vitro and in vivo models of glioma.^132^

The combination of HIF inhibition plus radiotherapy has been preclinically evaluated. Although the HIF inhibitors YC-1^121^ and PX-478^133^ can radiosensitise tumours in mouse xenografts, less toxic and more effective drug candidates are required to progress to clinical testing.

Together, these findings suggest that HIF inhibitors might still have potential for paediatric cancer treatment.

### HIF downstream target gene inhibition

VEGF plays a central role in regulating angiogenesis in hypoxic tumours, whilst GLUT1 and CAIX contribute to adaptations in hypoxic cancer cell metabolism.^18^ GLUT1 and CAIX are proteins located on the cell membrane with a functional role in glucose uptake and pH regulation, respectively. They increase glycolytic metabolism and enhance cell survival in low oxygen environments.^134^ These HIF downstream target genes can be inhibited, and have been investigated for therapeutic benefit to varying extents.

The combination of radiotherapy plus the anti-VEGF antibody bevacizumab has been investigated in a number of adult clinical trials.^135^ The radiation–bevacizumab combination is effective due to the temporary ‘normalising’ effect of VEGF inhibitors on the tumour vasculature: immediately after treatment, tumour perfusion and oxygen supply are temporarily restored due to the emergence of stable and more mature blood vessels, and pruning of immature and chaotic vessels conferring greater radiation-induced DNA damage.^136^ However, this restoration effect is followed by blood vessel regression, eventually leading to poor perfusion and a decrease in tumour oxygenation in tumours, during which time radiotherapy is less effective.^20^ The success of this combination relies on the development of suitable imaging and blood-based biomarkers to determine scheduling regimens as well as selecting suitable patients for treatment.^136^ Notably, combining VEGF inhibition with chemoradiotherapy in glioblastoma had no effect on OS in two Phase 3 trials, but resulted in more adverse effects. Patients in these studies were not pre-selected based on predictive markers.^137,138^

Bevacizumab is used in combination with chemotherapy or as a single agent in selected adult cancers.^139^ It demonstrated tolerability and safety in children in a Phase 1 trial in paediatric patients with refractory solid tumours^140^ and has since entered into Phase 2 trials. The Phase 2 BERNIE study in metastatic paediatric soft-tissue sarcomas concluded that the addition of bevacizumab to chemotherapy brought about no significant improvement in event-free survival.^141^ Similarly, bevacizumab in combination with irinotecan in paediatric malignant glioma and diffuse brainstem glioma failed to improve outcomes in children.^142^ However, in recurrent low-grade gliomas, the same combination produced sustained disease control in some children.^143^ The blood vessel regression caused by anti-angiogenic therapies can reduce perfusion levels, thereby limiting optimal drug delivery and further increasing tumour hypoxia.^144^ More research is required to determine which paediatric cancers might benefit from this combination.

Over the past 5 years or so, inhibitors of CAIX, such as SLC-0111, which sensitises several cancer cells lines to conventional chemotherapy, have been developed.^145^ SLC-0111 is being tested in a Phase 1/2 trial in combination with gemcitabine for pancreatic cancer (NCT03450018), and a Phase 1 trial for the treatment of advanced metastatic solid tumours was completed successfully.^146^ The potential of this treatment strategy is in the developmental stages, but considering that CAIX expression correlates with worse prognosis in certain childhood cancers,^24,25^ this approach could similarly be applied for treatment of paediatric cancers. Lastly, GLUT1 inhibitors, such as BAY-876^147^ and WBZ117, successfully restored radiosensitivity in radioresistant breast cancer cells.^148^

## Targeting hypoxia to improve the immune response in tumours

Increasing evidence supports the notion that hypoxia is immunosuppressive, decreasing the tumour innate and adaptive immune response.^149,150^ The resistance of various tumours to immunotherapeutic drugs could therefore be attributed to hypoxia. However, to date, the effect of tumour hypoxia on treatment response is only being evaluated in one immunotherapy trial (NCT03003637), in which adult patients are treated with nivolumab, a programmed cell death 1 (PD-1) inhibitor, in combination or not with ipilimumab, a cytotoxic T-lymphocyte-associated protein 4 (CTLA-4) inhibitor, in addition to standard of care.^151^

In an attempt to reverse the effects of hypoxia on anti-tumour immunity, the combination of immunotherapy plus hypoxia-targeted agents has been investigated. The benefit of targeting hypoxic cells with evofosfamide in combination with CTLA-4 and PD-1 checkpoint blockade was demonstrated in a transgenic adenocarcinoma mouse prostate model; this treatment resulted in a 30–50% increase in OS compared with single-agent treatment, with elevated levels of infiltrating T lymphocytes in hypoxic areas of tumours.^152^ An ongoing trial (NCT03098160) is investigating the combination of evofosfamide plus ipilimumab in several adult cancers.^153^ In a melanoma mouse model study, tumour growth was delayed, and survival was improved after combination treatment with SLC-0111 plus immune-checkpoint blockade.^154^

Immune-checkpoint inhibitors and chimaeric antigen receptor (CAR) T-cell therapy have been approved for the treatment of a subset of paediatric cancers.^155^ Whereas CAR T-cell therapy has produced some impressive results in this subset of patients, immune-checkpoint inhibitors have been less successful, possibly owing to the lack of neoantigens in paediatric cancers:^155^ as mentioned above, differences in the aetiology of adult and paediatric cancers exist—one example being the environmental induction of a high mutational burden in adult cancers that is largely absent from paediatric tumours.^156^

Immunotherapy for paediatric cancer patients is not without challenges^157^ although generally, fewer patients present with toxicities than those receiving more conventional therapies. However, the risk of severe and potentially fatal side effects remains and tends to be greater in children than in adults. For example, paediatric ALL patients appear to suffer from cytokine-release syndrome and neurological toxicities more frequently than adult ALL patients following treatment with CD19-targeted CAR T-cell therapy.^158^

Furthermore, hypoxia plays a major role in creating an immunosuppressive environment by stimulating the release of immunosuppressive metabolites.^159^ Alleviating hypoxia in paediatric cancers could therefore potentially create the necessary pro-inflammatory environment for immunotherapies to be effective. The combination of hypoxia-targeted treatments and immunotherapy remains untested in paediatric cancers, both preclinically and in clinical trials, as research in this field is in its infancy. Paediatric clinical trials will probably only follow successful adult clinical trials, and at this point, it is key that the level of hypoxia is evaluated as a potential prognostic or even predictive biomarker of response for immunotherapy treatment or combined hypoxia-targeted/immunotherapy approach, respectively.

## Overview and future perspectives

Adult cancer patients have benefited from being involved in clinical trials of hypoxia-targeted therapies; however, these treatments have largely been given in the absence of stratification by hypoxic status prior to enrolment—meaning little progress has been made, and these treatments are not commonly used in clinical practice. Importantly, as highlighted by Yang et al., studies that have prospectively or retrospectively stratified patients on the basis of hypoxia gene signatures have demonstrated that hypoxia-modifying treatments are more effective the more hypoxic the tumour is.^160^

To identify paediatric cancer patients that will benefit from hypoxia-targeted therapies, reliable biomarkers of hypoxia for children must first be developed. The benefit of novel hypoxia assessment techniques in children must outweigh the potential invasive nature of the technique. Three methods appear to be the easiest to integrate into routine diagnostic procedures. The endogenous marker osteopontin can be measured in patient blood samples, despite its limited specificity as a hypoxia marker. Furthermore, once validated for paediatric cancers, hypoxia gene signatures could be relatively easily evaluated in standard tumour biopsy samples. Novel IS- or OE-MRI imaging techniques, already implemented on standard clinical scanners, would allow for the longitudinal, minimally invasive assessment of tumour hypoxia levels without added radiation.

If patients can be systematically evaluated for the presence of hypoxia in their tumours, a few therapies might be translatable to children. Nimorazole, in combination with nicotinamide, has shown its immense therapeutic value as a radiosensitiser in HNSCC. Evofosfamide has been widely investigated in paediatric cancers as a HAP and, with biomarker-led clinical trials, it is likely to prove its clinical use in both adults and children. Finally, atovaquone, also a potential radiosensitiser, could be introduced relatively efficiently following its proven safety track record in children.

To protect children from unnecessary experimental treatments, it is essential that future clinical trials in children are based on sound rationale for the combination of hypoxia-targeted agents with existing treatments. This must be supported by robust preclinical data and inclusion of the least invasive and most informative techniques to stratify patient tumours according to their level of hypoxia.

## Data Availability

Not applicable.
